# Understanding the Therapeutic Potential of Ascorbic Acid in the Battle to Overcome Cancer

**DOI:** 10.3390/biom11081130

**Published:** 2021-07-31

**Authors:** Jurnal Reang, Prabodh Chander Sharma, Vijay Kumar Thakur, Jaseela Majeed

**Affiliations:** 1 Department of Pharmaceutical Chemistry, School of Pharmaceutical Sciences, Delhi Pharmaceutical Sciences and Research University (DPSRU), New Delhi 110017, India; jurnalreang026@gmail.com (J.R.); sharma.prabodh@gmail.com (P.C.S.); 2 Biorefining and Advanced Materials Research Centre, Scotland’s Rural College (SRUC), Kings Buildings, Edinburgh EH9 3JG, UK; 3 School of Allied Health Sciences, Delhi Pharmaceutical Sciences and Research University (DPSRU), New Delhi 110017, India

**Keywords:** cancer, ascorbic acid, anticancer, antioxidant, pro-oxidant, epigenetic regulator, HIF

## Abstract

Cancer, a fatal disease, is also one of the main causes of death worldwide. Despite various developments to prevent and treat cancer, the side effects of anticancer drugs remain a major concern. Ascorbic acid is an essential vitamin required by our bodies for normal physiological function and also has antioxidant and anticancer activity. Although the body cannot synthesize ascorbic acid, it is abundant in nature through foods and other natural sources and also exists as a nutritional food supplement. In anticancer drug development, ascorbic acid has played an important role by inhibiting the development of cancer through various mechanisms, including scavenging reactive oxygen species (ROS), selectively producing ROS and encouraging their cytotoxicity against tumour cells, preventing glucose metabolism, serving as an epigenetic regulator, and regulating the expression of HIF in tumour cells. Several ascorbic acid analogues have been produced to date for their anticancer and antioxidant activity. The current review summarizes the mechanisms behind ascorbic acid’s antitumor activity, presents a compilation of its derivatives and their biological activity as anticancer agents, and discusses delivery systems such as liposomes, nanoparticles against cancer, and patents on ascorbic acid as anticancer agents.

## 1. Introduction

**Figure 1:**
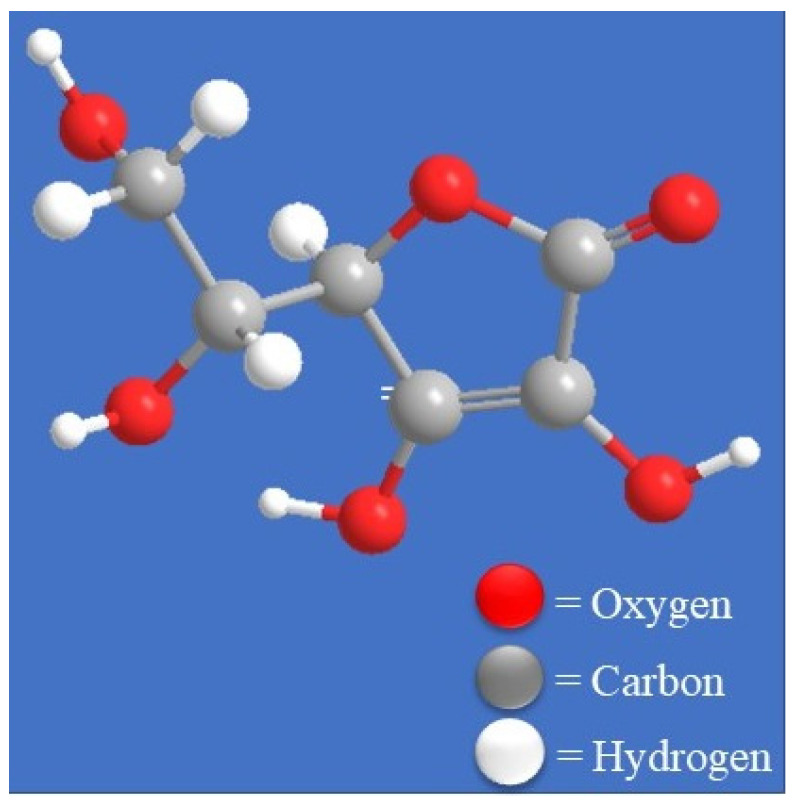
Chemical structure of ascorbic acid.

In 2018, the World Health Organization (WHO) estimated that 9.6 million people died from cancer, which is expected to cause 16.4 million deaths by 2040 [1,2]. Although there has been progress in cancer research to reduce mortality and improve the survival rate, cancer still accounts for nearly one in every six deaths and is the second leading cause of death worldwide [1]. There have been numerous approaches such as chemotherapy, radiotherapy and surgery to prevent and treat cancer, and some signs of progress have been made, but the need to develop pioneering, effective treatments for cancer still exists [3,4]. The prevalence of side effects and adverse drug reactions is the problem associated with existing cancer treatments [5]. The most common side effects are nausea, vomiting, hair loss, diarrhoea, anaemia, fatigue, and appetite and weight changes, and in long-term use, the treatments can cause permanent gastrointestinal dysfunction and damage to the reproductive system [6,7]. Thus, finding new and effective drugs with fewer side effects is necessary for cancer treatment. Ascorbic acid (Figure 1) is a vital micronutrient in our bodies, possessing antioxidant [8,9,10] and anticancer activity [8,10].

Humans are incapable of producing ascorbic acid in their bodies due to the absence of the enzyme gluconolactone oxidase, which is required for the generation of ascorbic acid from glucose and galactose [11]. Hence, it is essential for humans to take ascorbic acid through their diet from various sources to meet their physiological needs. Sources of ascorbic acid for human diets including the following.

### 1.1. Natural Sources

**Figure 2:**
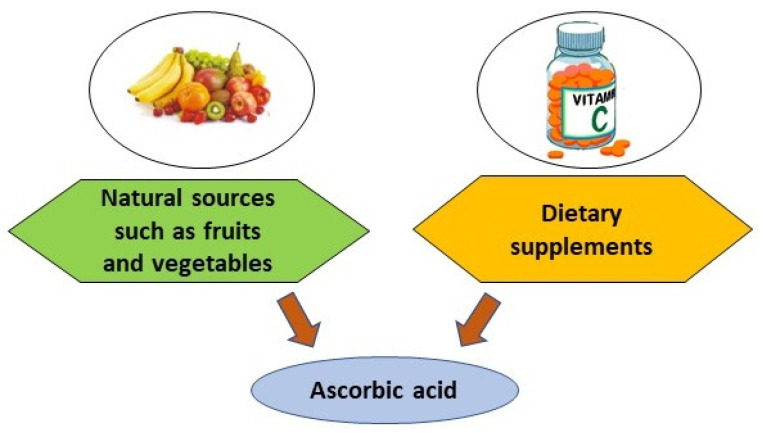
Sources of ascorbic acid.

Ascorbic acid is also known as vitamin C, which is widely found in citrus fruits, strawberries, tomatoes, broccoli, Brussels sprouts, green peppers, red peppers, turnips, and other leafy vegetables [12,13,14]; small amounts have also been found in fish and milk (Figure 2) [12].

### 1.2. Dietary Supplements

Ascorbic acid is available as a health supplement in different formulations such as powders for oral suspension, capsules, granules for oral solution, powders for oral solution, tablets, oral drops, and syrups in combination with proteins, omega-3 fatty acids, sugar, vitamin A, B1, B2, D, E, methylcobalamin (B12), pyridoxine (B6), niacin (B3), biotin (B7), pantothenic acid (B5), folic acid (B9), L-arginine, calcium, copper, iodine, iron, magnesium, phosphorus, potassium, selenium, sodium, and zinc (Figure 2) [15]. Sometimes, in dietary supplements, ascorbic acid may contain mineral ascorbates in the form of calcium ascorbate or sodium ascorbate [16]. 

Subject to dose and route of administration, ascorbic acid exhibits either antioxidant or anticancer activity; at lower concentrations, it functions as an antioxidant, and at higher pharmacological concentrations, as a pro-oxidant [10]. 

Ascorbic acid plays the role of an antioxidant agent by reducing unstable free radicals of oxygen, nitrogen, and sulphur and also by enhancing the activity of another antioxidant, tocopherol (vitamin E) [17]. Thus, ascorbic acid protects DNA, amino acid residues, and lipids from oxidation induced by free radicals and maintains their integrity [18], preventing them from harmful mutations. At pharmacological concentrations, ascorbic acid generates H_2_O_2_ in cancer cells [19] through organometallic reaction [20] and causes selective cytotoxicity to cancer cells [19]. About 90% of cancer deaths are rooted in metastasis; in metastasis, cancerous cells are spread from the primary tumour site to surrounding tissues and distant organs through the blood and lymphatic vessels [21,22,23]. Cancer becomes highly fatal and incurable after spreading beyond the primary tumour site [21]. In in vitro study, it was observed that ascorbic acid at high concentration curbed cell migration and capillary-like tube formation [24]. Accordingly, ascorbic acid can be useful to prevent the further growth and spread of tumour cells. Moreover, ascorbic acid not only protects our body from free radicals as an antioxidant and damages cancerous cells as a pro-oxidant, but it is also involved in many important physiological functions such as the formation of collagen, wound healing, repair of body tissues, and nurturing of bones, cartilage, and teeth [25]. In addition, ascorbic acid is less toxic towards the normal cells [26] and it is cheap and easily available; therefore, it could be an ideal agent to develop an anticancer agent. This up-to-date review focuses on the importance of ascorbic acid to improve cancer remedies. It focuses on recent advances and reviews the biological activity of various ascorbic derivatives as anticancer and antioxidant agents on different cell lines.

## 2. Metabolism

**Figure 3:**
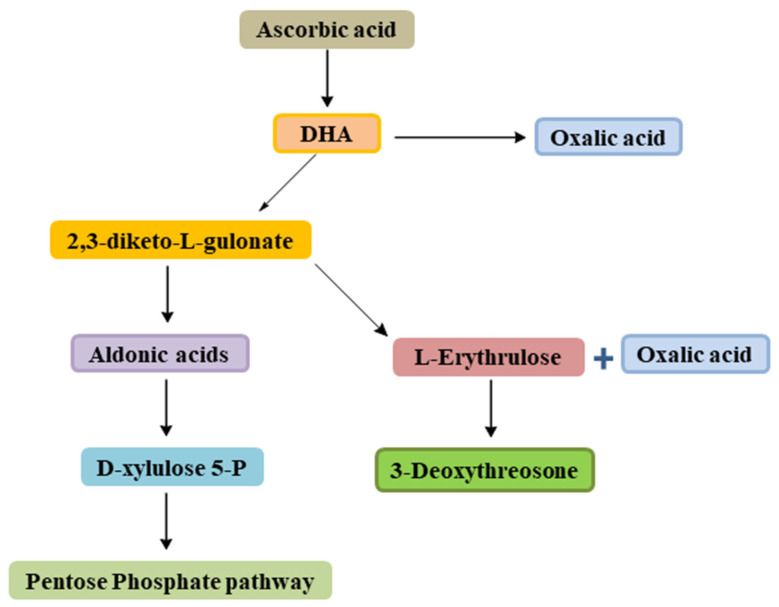
Degradation of ascorbic acid into various products during metabolism.

Physiologically, ascorbic acid exists in L- ascorbic acid and L-dehydroascorbic acid configurations [12]. On oxidation, L-dehydroascorbic acid irreversibly gives 2, 3-diketo-L-gulonic acid [12,13,27], which is then converted to C5 aldonic acids followed by D-xylulose 5-P; later enter in key metabolism through the pentose phosphate pathway. Also, 2, 3-diketo-L-gulonic acid is also converted to L-erythrulose, which next is promoted to 3-deoxythreosone [27]. During metabolism, due to its complex chemical nature, L-dehydroascorbic acid can produce several products that may contribute to body physiology and also to disease pathology, including 3- deoxythreosone, a reactive agent that glycates lens proteins, and oxalates that contribute to the development of kidney stones by forming calcium oxalate (Figure 3) [27]. The metabolites of ascorbic acid are primarily eliminated through urine [13]. It is pertinent to mention here the SVCT role in mediating the transport of ascorbate into the cell for activation [28]. 

## 3. Structural Features 

**Figure 4:**
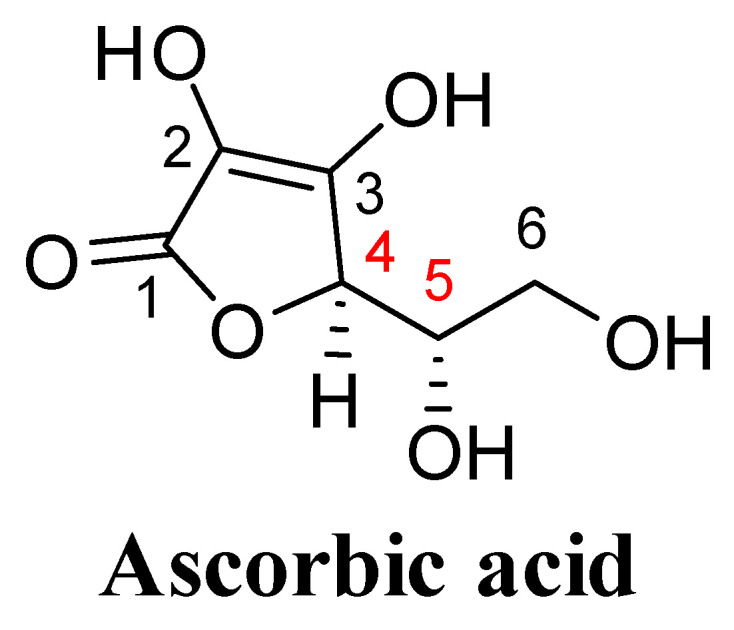
Numbering of the different carbon atom of L-ascorbic acid (4R, 5S).

Ascorbic acid is a five-membered gamma-lactone ring sugar acid, containing four hydroxyl groups at different positions. The C2 and C3 hydroxyl groups of L-ascorbic acid linked with its C1 carbonyl group make the proton on the C3 hydroxyl group suggestively acidic (pK1 = 4.25) in comparison to the C2 hydroxyl group proton (pK2 = 11.79). At physiological pH, it exists in the form of ascorbate anion, which is a good reducing agent that is oxidized into ascorbate free radical and later into dehydroascorbate [29,30]. The C5 and C6 hydroxyl groups are typical alcohol groups that form acetals and ketals on reaction with aldehydes and ketones, respectively. The two asymmetric centres at C4 and C5 of L-ascorbic acid are responsible for positive optical rotation (Figure 4) [29]. The two asymmetric centres are also responsible for different diastereoisomers such as L-ascorbic acid (4R, 5S), D-ascorbic acid (4S, 5R), L-isoascorbic acid (4R, 5R), and D-isoascorbic acid (4S, 5S).

## 4. Possible Interventions in Cancer Treatment

**Figure 5:**
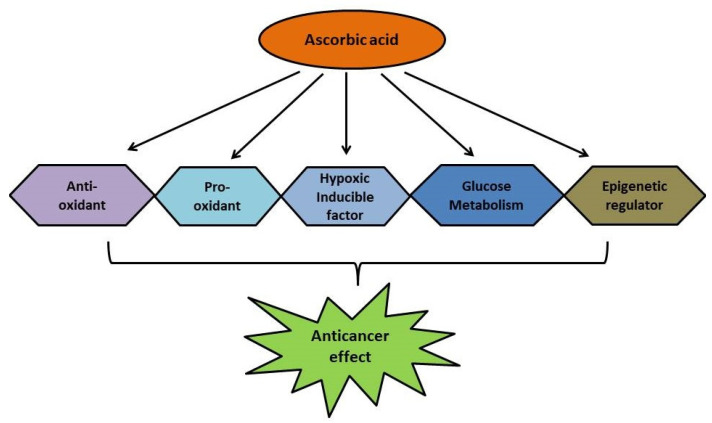
The different mechanisms involved in the interventions of ascorbic acid as an anticancer agent.

In 1976, when Ewan Cameron and Linus Pauling reported that ascorbic acid administration to cancer patients increased their survival rates [31], ascorbic acid drew significant attention for its potential to possess anticancer activity. Subsequently, several studies have been approved to assess the anticancer potential of ascorbic acid. In cancer, the affected cells further invade normal cells in different parts of the body. Cancer cells have several aids for growth and invasion: namely, glucose transporters (GLUTs), hypoxia-inducible factor (HIF), and ten-eleven translocation (TET) proteins. GLUTs transport glucose to cancer cells, enhancing growth and invasion (Figure 5). HIF controls the expression of genes linked to angiogenesis, anti-apoptotic activity, stem cell renewal, metastasis, and therapeutic resistance of cancer cells. TET proteins are associated with the activation of cancer stem cells by altering the metabolic and epigenetic profiles of cells. Finding novel characteristics of ascorbate with pathways it controls in cancer will assist in the selection and development of innovative therapies. In light of its anticancer potential, studies support that ascorbate is linked to the crucial functions of the GLUT, HIF, and TET mechanisms. From different studies, it was observed that ascorbic acid combats cancer by the following mechanisms of action:

### 4.1. As the Down Regulator of Hypoxic Inducible Factors

**Figure 6:**
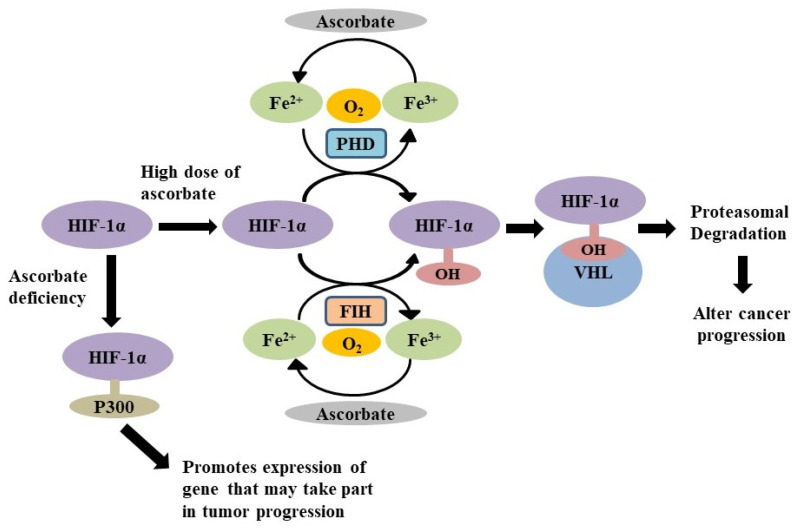
Ascorbate regulates HIF-1α by recycling Fe^2+^ required for the function of PHDs and FIH, leading to alteration of cancer progression [34,35,37,41,42].

Oxygen deficiency with severe hypoxia has been observed in cancer patients [32]. The constant O_2_ deprivation in cells induces stimulation of hypoxia-inducible factors (HIF) [33,34], which transform the expression of numerous genes, resulting in angiogenesis and development of erythropoietic stem cells [33]. In normoxic environments, HIF expression is strongly controlled by special oxygen-responsive prolyl hydroxylase domain enzymes (PHDs); these enzymes hydroxylate the proline residues of HIFs and regulate HIF activity [32,33,34,35]. In addition, under normoxic conditions factor inhibiting HIF (FIH) also regulates HIF-1α activity through hydroxylating asparagine residues of HIF-1α by obstructing the recruitment of coactivator p300, which leads to transcriptional inactivation of HIF-1α [36]. The PHD-induced hydroxylation process is very important, as it is involved in the proteasomal destruction of ubiquitinated HIF-1α by von Hippel-Lindau (VHL) protein [34,35,37]. The VHL protein identifies hydroxylated HIF and binds to it, then accumulates the ubiquitin ligase, resulting in cleavage of HIF-1α [37,38]. Because the PHD and FIH use oxygen as a substrate, under hypoxic conditions the process cannot take place [39], leading to activation of HIF. PHDs are a class of dioxygenases, requiring iron and 2-oxoglutarate to exert activity [34,40]. Ascorbate acts as a co-factor to the PHDs to recycle and maintain ferrous ion (Fe^2+^) [40,41]. Therefore, the presence of ascorbate can impact HIF function, which in turn can affect tumour progression [40]. Wilkes et al., have revealed that pharmacological ascorbate induced degradation of HIF-1α, leading to decreased metastatic development in pancreatic cancer [42]. At higher doses, ascorbic acid reduces DNA damage and mutations through degradation of HIF-1α levels (Figure 6) [43]. This suggests that ascorbate at higher doses in cancer cells inhibits HIF-1α activity by enhancing the action of HIF hydroxylases, thus suppressing tumour growth.

### 4.2. Impairing Glucose Metabolism in Cancer Cells 

**Figure 7:**
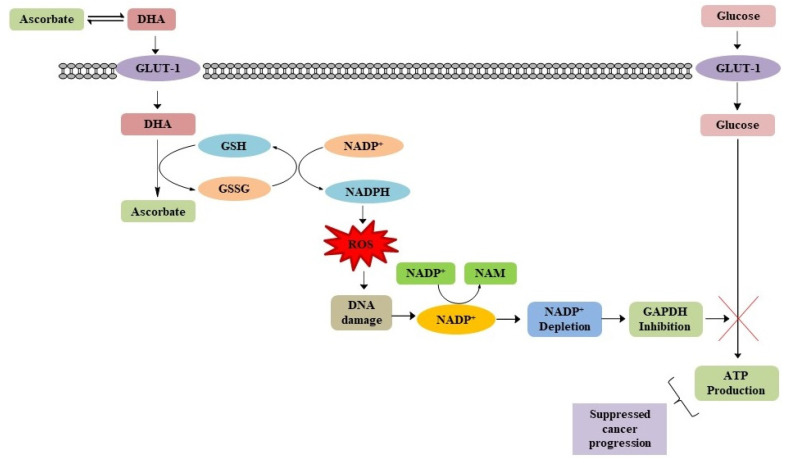
Role of ascorbate in impairing glucose metabolism in cancer cells [41,46,51,52,53].

In the early 1900s, Otto Warburg and co-workers observed that a higher amount of glucose was consumed for glycolysis by tumour cells in the absence of abundant oxygen; this is known as the Warburg Effect [44]. It is a crucial characteristic of tumour survival and proliferation in hypoxic environments [45]. This change in glycolytic character allows tumour cells to undergo 200 times faster rates of glycolysis than normal cells [46]. The Warburg Effect is a vital metabolic trait of cancer cells [47]. Yu et al., have observed that mutations of KRAS or BRAF genes in cancer cells upregulate glucose transporter-1(GLUT1) genes promoting the Warburg effect [48]. Under low glucose conditions, cancer cells survive through increased glycolysis resulting from selective upregulation of GLUT1, which could be a potential approach to treat cancer [49]. It was perceived that intravenous high concentrations of ascorbic acid selectively interfere with mutated KRAS or BRAF, inducing GLUT1 regulation of glycolysis and killing cancer cells. After administration into the body, ascorbic acid oxidizes to dehydroascorbate (DHA) [50]. Due to its structural similarity to glucose, DHA is taken up by KRAS or BRAF mutated GLUT1 in cancer cells [41]. Once it enters the cell, DHA in the presence of nicotinamide adenine dinucleotide phosphate (NADPH) and glutathione (GSH) is reduced back to ascorbic acid [50]. In this reduction process, the alteration of GSH and NADPH to glutathione disulphide (GSSG) and oxidized nicotinamide adenine dinucleotide phosphate (NADP+), respectively, gives rise to cellular ROS [50,51]. The raised ROS level damages DNA guiding activation of poly (ADP-ribose) polymerase (PARP), which depletes nicotinamide adenine dinucleotide (NAD+), an important cofactor for the conversion of glyceraldehyde 3-phosphate dehydrogenase (GAPDH) to nicotinamide (NAM). This hindrance of GAPDH in KRAS or BRAF mutant cells leads towards scarcity of energy followed by cell death as mitochondria produce less ATP [50]. In gastric and renal cancer, it was observed that there is high expression of GLUT1 with higher glycolytic activity and that they are sensitive to high doses of ascorbate treatment, resulting in the selective killing of cancer cells (Figure 7) [52,53]. Hence, ascorbic acid can induce toxicity in cancer cells by impairing glucose metabolism.

### 4.3. As an Epigenetic Regulator 

**Figure 8:**
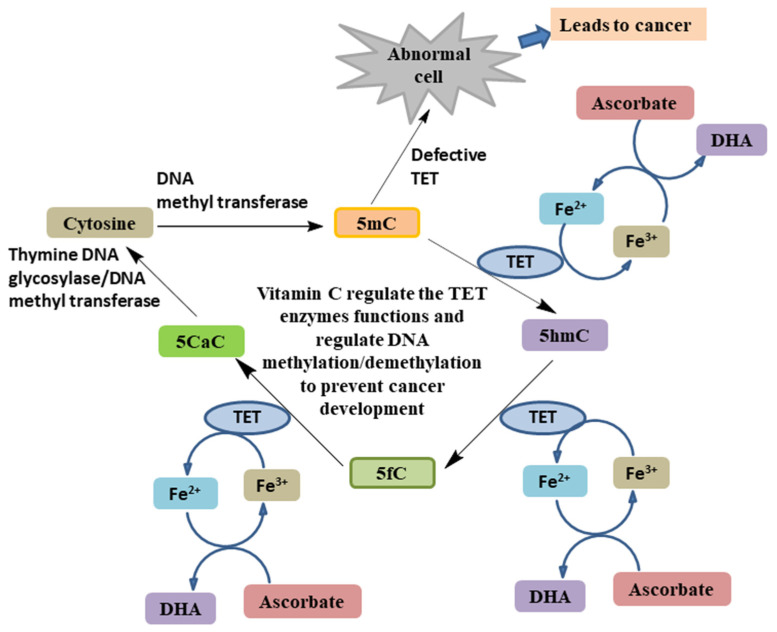
Role of ascorbate in the regulation of TET enzymes [41,59,60,65].

Altered genetic and epigenetic profiles caused cancer and cancer progression [54]. In epigenetic modifications, there are not any direct variations in the DNA sequence; rather, there is an array of gene expression such as DNA methylation/demethylation [55]. Hypomethylation triggers various intranuclear transcription factors by which it promotes cancer formation [56], and hypermethylation facilitates tumorigenesis by confining promotors of tumour suppressor genes [30,56,57]. Mutation and altered expression of ten-eleven translocation (TET) protein families [45] lead to abnormal DNA methylation, which contributes to cancer formation [58]. In hematopoietic malignancies, TET (TET2) is commonly mutated [58]. TET proteins are iron/α-ketoglutarate-dependent dioxygenase (Fe^2+^/α-KGDD) enzymes [59]; the family comprises TET1, TET2, and TET3 proteins [58]. These enzymes require cofactor ascorbic acid to exert their activity [18,60]. TET proteins catalyse the conversion of 5-methylcytosine (5mC) to 5-hydroxymethylcytosine (5hmC), which in later steps aids oxidation to aldehyde to form 5-formylcytosine (5fC) and carboxylic acid to form 5-carboxylcytosine (5CaC) [61,62]. Oxidized 5fC and 5CaC undergo base excision mechanism in the presence of thymine DNA glycosylase enzyme and are transformed to cytosine [63]. Throughout the enzymatic activity of TETs, ascorbate acts as a cofactor to promote the recovery of Fe^3+^ to Fe^2+^ (Figure 8) [64]. Different types of haematological malignancies such as acute myeloid leukaemia (AML) and chronic myelomonocytic leukaemia (CMML) are associated with mutation-led hindered catalytic activity of TET2 [65]. Shenoy et al., have observed that ascorbic acid in lymphoma patients modifies TET function by increasing the suppression of tumour suppressor gene SMAD1 and improved chemosensitivity [60]. Ascorbic acid in AML cells and TET2 deletion mouse models induces DNA hypomethylation and elevated TET activity [59]. Thus, TET enzymes block significant protagonists in cancer progression and ascorbate accessibility impacts their activity.

### 4.4. As an Antioxidant Agent 

**Figure 9:**
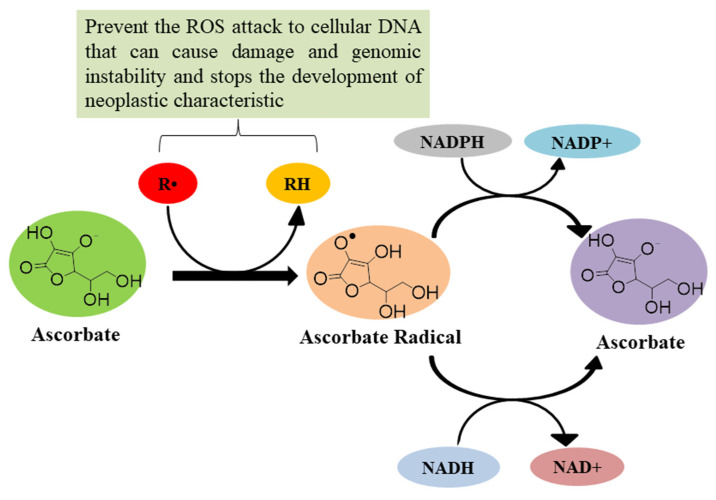
Antioxidant mechanism of ascorbic acid [40,41,71].

In the human body, reactive oxygen species (ROS) are generated during physiological and pathological processes [66,67], but high levels of ROS contribute to carcinogenesis [68]. ROS attack cellular DNA, causing damage and genomic instability and leading to mutations that incorporate the development of neoplastic characteristics [69,70]. In healthy humans, ascorbate at 40–80 μM of plasma concentration exerts antioxidant activity; at this concentration, ascorbate donates an electron to free radicals and diminishes their potential damaging effects [30]. During this process, ascorbate is itself oxidized into a fairly unreactive ascorbate radical, which through NADH/NADPH-dependent reductases is converted back to ascorbate (Figure 9) [30]. Ascorbate in association with vitamin E synergistically functions as a co-antioxidant to shield LDL from ROS-induced lipid peroxidation [30] and prevent the formation of the end-product of the process, 4-hydroxynonenal (HNE), which is considered as a secondary messenger of oxidative stress [45]. Ascorbate also maintains vitamin E levels in the body after the inhibition of lipid oxidation [71]. In addition, ascorbic acid supplementation as an antioxidant during chemotherapy increases tumour response to treatment and also increases survival rate [72]. Suh et al., have observed that ascorbate in vitro acts as an anti-oxidant by preventing iron-induced lipid peroxidation [73]. Chen et al., have reported that orally supplemented ascorbate in guinea pigs with iron overload acts as an antioxidant by suppressing lipid oxidation [74]. Thus, ascorbic acid at lower concentrations acts as an antioxidant to prevent damage caused by free radicals to the body and also prevents degradation of vitamin E by enhancing the effects of chemotherapy.

### 4.5. Pro-Oxidant Role 

Ascorbic acid at micromolar (μM) concentrations functions as an anti-oxidant agent, but at higher, millimolar (mM) concentrations, also functions as a pro-oxidant. Intravenous administrations of vitamin C induce cytotoxicity to tumour cells [75], as it can produce 70-fold greater plasma concentration than its oral counterpart [76]. It was observed that the antitumor potential of high-dose ascorbic acid is rooted in its ability to generate hydrogen peroxide (H_2_O_2_) [77]. In animals, ascorbate at millimolar concentrations donates an electron to copper and iron metals, resulting in the production of superoxide, a hydrogen peroxide-like ROS [20]. The ROS induces damage to DNA, ultimately causing cytotoxicity to cancer cells [55]. Upon intravenous administration, ascorbate (AscH−) reacts with protein-centred transition metal ions, i.e., ferric (Fe^3+^) and cupric (Cu^2+^) ions. In practice, there is a fairly low level of conversion metals, and thus greater concentrations of vitamin C are necessitated. Ascorbate reduces Fe^3+^/Cu^2+^ to ferrous (Fe^2+)^/cuprous (Cu+) ions, oxidizing itself into ascorbate free radical (Asc−). The reduced Fe^2+^/Cu+ ions react with oxygen to form ROS such as superoxide radicals, which in the existence of hydrogen undergo dismutation and form hydrogen peroxide (H_2_O_2_). Further, the H_2_O_2_ may undergo a Fenton-like reaction catalysed by Fe^2+^/Cu+, yielding hydroxyl peroxide radical (HO⋅) [30,55], which induces damage to cancer cells [78].

AscH− + Fe^3+^/Cu^2+^ → Asc•− + Fe^2+^/Cu+

Fe^2+^/Cu+ + O_2_ → Fe^3+^/Cu^2+^ + O_2_•−

O_2_•^−^ + O_2_•− + 2H+ → H_2_O_2_ + O_2_

Fe^2+^/Cu+ + H_2_O_2_ → Fe^3+^/Cu^2+^ + HO•

It was reported that tumour tissues contain proteins in interstitial fluid [79] and the damaged protein in extracellular fluid contains catalytic metals such as iron and copper [80]. Compared to normal endothelium, tumour vessels have greater permeability [79], which amplifies the metal ion required for catalytic conversion and enhances the transformation of ascorbic acid to H_2_O_2_ [80]. Several studies have reported elevated copper levels in cancer [81,82], as it is a cofactor of DNA replication enzymes for rapidly proliferating cancer cells [55]. In cancer, elevated copper concentrations with an altered systemic distribution of the element [83] made cancer cells vulnerable to the ROS-generated selective cytotoxicity of copper and ascorbic acid [84]. Additionally, in the case of iron, although it is stored by the glycoprotein ferritin in healthy individuals, under pathological conditions such as cancer inflammation, extracellular iron chelates are increased in tissue [85], making them susceptible to ascorbate-induced ROS toxicity.

Chen et al., have observed that ascorbic acid selectively hinders tumour growth without causing any damage to normal cells via the generation of H_2_O_2_ [19]. Baek et al., also observed that ascorbic acid induces toxicity to cancer cells through the generation of ROS [86]. In another study, a high quantity of ascorbic acid prompted the cell death of malignant cells through the generation of ROS, especially hydrogen peroxide [87]. Thus, ascorbic acid at millimolar concentrations induces toxicity to various cancer cells through the generation of ROS, which themselves damage cellular components and hamper various important cellular mechanisms. 

## 5. Potent Synthetic Derivatives of Ascorbic Acid against Cancer

L-ascorbic acid possesses few susceptible sites available for substitution, generating a sum of compounds with of exciting chemical, physical, and biological characteristics. Previous accounts of ascorbic acid analogues substituted to C2, C3, C5, and C6 positions have been reported in various studies [88,89]. Designing new ascorbic acid analogues as anticancer agents follows derivatization on any of the above-mentioned sites and the evaluation of their activity against some of the following cell lines, such as hepatocellular carcinoma (HepG2), colorectal adenocarcinoma (SW620), lung adenocarcinoma (A549), colorectal carcinoma (HCT-116), breast adenocarcinoma (MCF-7), cervical carcinoma (HeLa), ductal pancreatic adenocarcinoma (CFPAC-1), murine colon carcinoma (Colon-26), murine leukaemia (L1210), pancreatic carcinoma (MiaPaCa-2), acute lymphoblastic leukaemia (CEM), Burkitt’s lymphoma (Raji line), myeloma (CCL155), colon cancer cells (HT29), hepatoma cells (HuH7), prostate cancer line (PC3), and non-small cell lung carcinoma (H-460). Following are some of the ascorbic acid analogues evaluated as antitumor and antioxidant agents.

**Figure 10:**
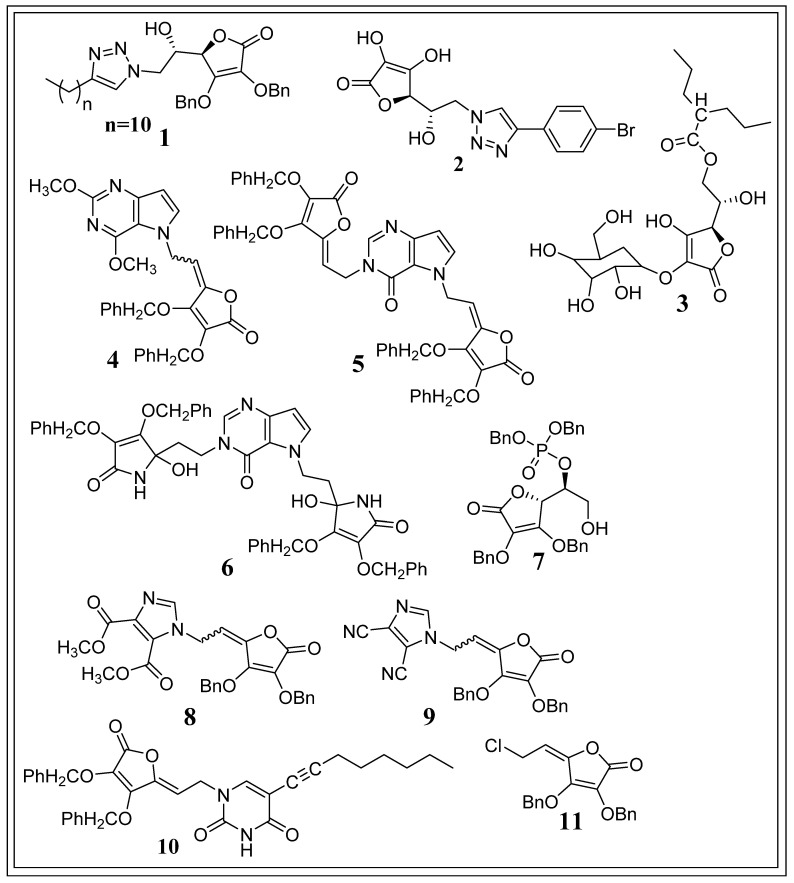
Ascorbic acid analogues showing antitumor effects.

Macan et al. (2019) synthesized two sequences of 6-(1,2,3-triazolyl)-2,3-dibenzyl-L-ascorbic acid analogues. Newly synthesized 6-(1,2,3-triazolyl)-6-deoxy-L-ascorbic acid and 6-(1,2,3-triazolyl)-4,5-didehydro-5,6-dideoxy-L-ascorbic acid analogues were assessed for antiproliferative activity. Among all the synthesized derivatives, compound **1** (Figure 10) (2,3-O, O-dibenzyl-6-(4-decyl-1,2,3-triazol-1-yl)-6-deoxy-L-ascorbic acid) displayed strong cytostatic activity, predominantly against breast adenocarcinoma cell lines (IC_50_ = 0.08 µM), while causing no toxicity to normal fibroblasts (IC_50_ > 100 mM). Compound **1** induces cytotoxicity through regulation of the HIF-1α signalling pathway [90].

Harej et al. (2019) synthesized novel 4-substituted 1,2,3-triazole L-ascorbic acid derivatives with hydroxy ethylene linkers and assessed their antiproliferative action. Among all the synthesized derivatives, compound **2** (Figure 10) (6-[4-(4-bromophenyl)-1,2,3-triazol-1-yl]-6-deoxy-L-ascorbic acid) showed the highest selectively antiproliferative activity towards breast adenocarcinoma cells (IC_50_ = 6.72 µM). In addition, compound **2** was not cytotoxic towards foreskin fibroblasts and showed little cytotoxicity towards lung fibroblasts (IC_50_ = 73.93 µM). Compound **2** (IC_50_ = 6.72 µM) showed cytotoxicity higher than that of reference compound carboxyamidotriazole (IC_50_ = 14.69 µM) but lower than that of reference compound 5-fluorouracil (5-FU) (IC_50_ = 0.096 µM) against MCF-7 cell lines. It was revealed that compound **2** exhibited cytotoxicity against MCF-7 cells through HIF-1-triggered hypoxia [91]. 

Miura et al. (2018) synthesized 2-O-α-D-Glucopyranosyl-6-O-(2-Pentylheptanoyl)-L-ascorbic acid (compound **3**) (Figure 10) and evaluated its antitumor activity on cells and tumour-bearing animal models. It was reported that the synthesized derivative did not show cytotoxicity against colon-26 cells at a 2 mM concentration. However, when administered at 1.7 mmol/kg of intravenous (IV) doses on every alternate day for 4 times, compound **3** inhibited tumour progression more strongly than control substances ascorbic acid and 2-O-α-D-Glucopyranosyl-L-ascorbic acid, a steady ascorbic acid derivative, despite comprising only 10 per cent of the molar amount of the control substance dose. This study suggested that compound **3** after administration converted to its metabolite AA-2G, which later oxidized to DHA and selectively killed the cancer cells through induction and increased endogenous oxidative stress [92]. 

Babic et al. (2015) synthesized new halogenated 3-deazapurine, 7-deazapurine, alkylated 9-deazapurine analogues of L-ascorbic acid and imino-L-ascorbic acid. The newly created derivatives were assessed for antitumor activity, and it was observed that, among all synthesized analogues, compound **4** (Figure 10), a 9-deazapurine analogue of L-ascorbic acid, showed the highest inhibitory activity towards CEM cells (IC_50_ = 4.1 ± 1.8 µM) and strong inhibitory activity against L1210 cells (IC_50_ = 4.7 ± 0.1 µM). Additionally, compound **5** (Figure 10) a 9-deazahypoxanthine analogue of L-ascorbic acid, demonstrated the highest antiproliferative activity against HeLa cells (IC_50_ = 5.6 ± 1.3 µM) and robust inhibitory activity against L1210 cells (IC_50_ = 4.5 ± 0.5 µM), whereas compound **6** (Figure 10), a disubstituted 9-deazapurine analogue with two imino-L-ascorbic acid moieties, exhibited the strongest inhibitory activity towards L1210 cells and MiaPaCa-2 cells (IC_50_ = 4.4 ± 0.3 µM, IC_50_ = 5.7 ± 0.2 µM, respectively). In addition, compounds **4**, **5**, and **6** were not cytotoxic towards 3T3, a murine embryonal fibroblast cell (IC_50_ > 100 µM), as compared to the standard drug, 5-fluorouracil (28.3 ± 0.01 µM) [93]. 

Bordignon et al. (2013) synthesized ascorbic acid derivatives by addition of phosphate or adenine side chains to the furanic ring, yielding a structure similar to ATP, and evaluated their antiproliferative activity. Among the synthesized derivatives, it was observed that compound **7** (Figure 10) (dibenzyl (S)-1-[(R)-3,4-bis(benzyloxy)-5-oxo-2,5-dihydrofuran-2-yl]-2-hydroxyethyl phosphate) exhibited the highest cytotoxicity towards HuH7, HT29, Raji and CCL155 cell line (IC_50_ = 0.1 ± 0.002, 0.15 ± 0.011, 0.086 ± 0.004 and 0.1 ± 0.005 mM, respectively) without causing any cytotoxicity against normal human cells. Compound **7** was further studied on xenografted animals with two dissimilar human tumour cell lines, HT29 and PC3. At 10 mg/kg/d dose, compound **7** on PC3 xenografted mice notably lowered tumour development and tumour weight, leading to the survival of all compound **7** treated mice at the termination of the study, whereas only 6 of 10 placebo-treated mice remained alive. The researchers also pointed out that compound **7** inhibited tumours by suppressing the expression of translation initiation factor and tRNA synthetase [94]. 

Wittine et al. (2012) synthesized new 1,2,4-triazole L-ascorbic acid, imidazole L-ascorbic acid, and imino-ascorbic acid derivatives and assessed their antitumor properties. Among all synthesized derivatives, compounds **8** and **9** (Figure 10) exhibited the greatest evident cytostatic effects towards entirely used tumour cell lines, and were selectively cytotoxic against CEM/0 cells (IC_50_ of 10 ± 4 and 7.3 ± 0.1 µM, respectively). Although compound **9** was more active than compound **8**, it showed no cytotoxicity towards human normal fibroblasts (IC_50_ > 100 µM) unlike compound **8** (IC_50_ = 73 ± 0.5 µM). Compound **8** and **9** were both less cytotoxic than the reference drug doxorubicin (IC_50_ = 0.39 ± 0.21 µM) towards CEM/0 cells but more than another reference drug, ribavirin (IC_50_ = 63 ± 14 µM). They also observed that compound **8** exhibited an antitumor effect through inhibition of inosine 5′-monophosphate dehydrogenase (IMPDH) [95].

Gazivoda et al. (2007) synthesized two series of new C-5 alkynylated pyrimidine and fused bicyclic furo[2,3-d]pyrimidine analogues of ascorbic acid. The newly synthesized compounds’ cytostatic activities were evaluated, and it was observed that from all the synthesized derivatives, compound **10** (Figure 10) showed the highest cytostatic activity against Molt4/C8, CEM, MiaPaCa-2, SW 620, MCF-7, and H-460 cell lines (IC_50_ = 3.0 ± 1.1, 2.0 ± 0.3, 3 ± 0.5, 4 ± 0.3, 4 ± 1.4, and 2.4 ± 0.4 µM, respectively) [96].

Gazivoda et al. (2006) synthesized 5,6-di-O-modified L-ascorbic acid analogues and evaluated their cytostatic activity. It was reported that among all synthesized compounds, Z-2,3-Di-O-benzyl-6-chloro-4,5-didehydro-L-ascorbic acid (compound **11**) (Figure 10) exhibited prominent antitumor activity against all the malignant tumour cells with an IC_50_ of 18 ± 1 µM, while also exerting cytotoxicity towards human normal diploid fibroblasts cells (WI 38) (IC_50_ = 26 ± 1 µM) [97].

**Figure 11:**
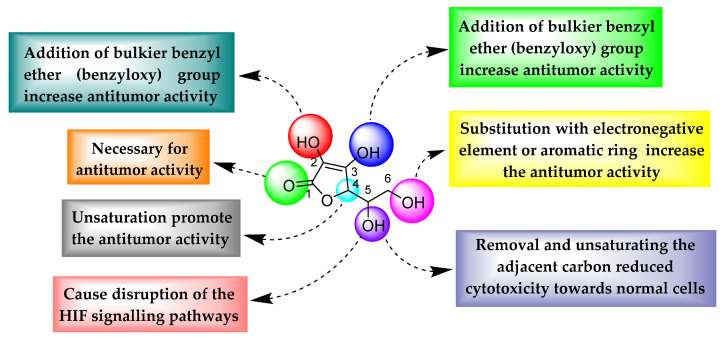
Important structural features of ascorbic acid analogues showing antitumor effects.

From all of the above derivatives, it was observed that protecting 2,3-hydroxyl groups with bulky benzyl ether (benzyloxy) groups decreased polarity, which in turn promoted the antitumor activity of the derivatives. The C1 carbonyl oxygen is essential to exert antitumor effects. In the case of compounds **4**, **5**, **8**, **9**, **10**, and **11** (Figure 10), it was observed that unsaturation of the C4 carbon further increased antitumor activity. At the 6th position, substitution with an electronegative element or aromatic ring increased the antitumor activity. Substitution or alteration with an electron-withdrawing group at positions 2, 3, 5, and 6 is vital for the antitumor potential of ascorbic acid (Figure 11).

## 6. Emerging Trends in Cancer Therapy 

As a demand to enhance the effects of treatment, the integration of molecules into different encapsulated delivery systems is important, as it protects the molecule from chemical breakdown [98]. Various studies have displayed successful and effective integration of ascorbic acid and its analogues into encapsulated delivery systems; some of these are described below:

### 6.1. Liposomes 

**Figure 12:**
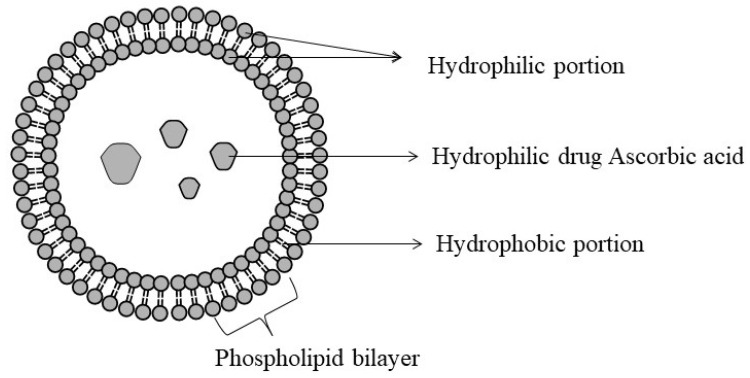
Structure of liposome.

Liposomes are biodegradable, small, artificial, spherical vesicles created from natural or synthetic phospholipids and cholesterol [99,100]. The phospholipids and cholesterol that make up liposomes are amphipathic, consisting of a hydrophilic head and lipophilic tail [100,101]. The polar heads and the hydrophobic tails form a bilayer that aids them in entrapping lipid-soluble and water-soluble ingredients in the hydrophobic and hydrophilic spheres, respectively [100]. Thus, owing to hydrophobic and hydrophilic characteristics [99] they can tweak the pharmacokinetic characteristics of drugs, herbs, and proteins (Figure 12) [100].

Filipczak et al., developed liposomes composed of ammonium ascorbate, mitoxantrone (MTX), and anacardic acid. The invented liposomal preparations selectively magnified the level of apoptosis in melanoma cell lines through specific production of free radicals by an iron ion mechanism. Morphological studies revealed a consistent circular shape with diameters spanning between 102 to 120 nm. The liposomal entrapped drugs are less toxic towards Hep-G2 and H9C2 cell lines in comparison to free drugs. After 12 h of administration of liposomes, the caspase activity was increased eight-fold against A375 melanoma, which led to generation of excessive reactive oxygen species, resulting in cell death [102]. 

Yang et al., developed liposomes of palmitoyl ascorbate (PA) with doxorubicin (DOX). In some classes of cancer therapy, PA diminishes DOX-triggered toxicity. The liposomes at high concentrations generate an increased quantity of ROS production and lead to the initiation of apoptosis. Morphological studies confirmed that the liposomes were spherical and homogeneous with a diameter ranging from 91 to 137.5 nm. Because PA prolonged plasma half-life, in vivo, it helps in extended drug retention. The liposomes containing PA and doxorubicin reduced the weights and sizes of the tumour by 2.5-fold and 5-fold compared to liposomes containing DOX and DOX in solution, respectively [103].

Li et al., developed a liposome co-delivery system of palmitoyl ascorbate (PA) with docetaxel (DC) for antitumor therapy. Synergistically, PA was used in combination with cytotoxic chemotherapy (DOC) to enhance its efficacy and cytotoxic activity. The drug-loaded liposomes were uniform, with particle sizes ranging between 140 to 170 nm. In in vitro tests, the liposomes consisting of PA and DC at a 200:1 weight ratio exerted maximum synergistic effects towards MCF-7, PC-3, and HepG2 cell lines. In addition, they inhibited tumour cell progression in vivo more efficiently than liposomes consisting of either drug alone [104]. 

Lipka et al., developed a coencapsulated epirubicin (EPI) liposome with ascorbic acid and ammonium ascorbate salt gradient. EPI, an antineoplastic agent, inhibits DNA and RNA synthesis by hindering topoisomerase II and is also liable for free radicals, resulting in toxicity against cancer cells. The drug-loaded liposomes are uniform with particle sizes ranging from 112 to 123 nm. The plasma abolition of free EPI is very fast compared to the liposome-encapsulated drug. In the case of the free drug, only 1% remains present in the plasma after 15 minutes, whereas for the encapsulated drug, about 40% is still present even after 24 hours. The liposomal encapsulated EPI with ascorbic acid gradient exhibited impressive antitumor activity in vivo against a breast cancer 4T-1 murine model [105].

### 6.2. Nanoparticles 

**Figure 13:**
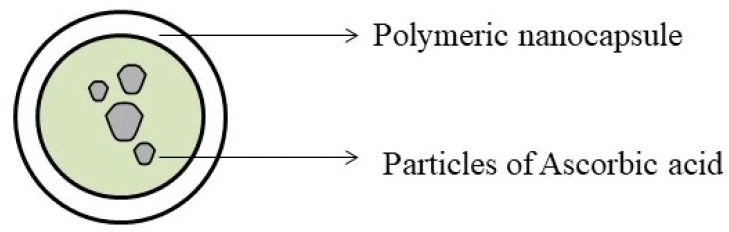
Nanoparticles containing drug particles.

Nanoparticles (NPs) (Figure 13) are particles made up of a wide range of materials usually with particle sizes ranging from 1 to 100 nm [106,107]. Researchers have observed that the physiochemical properties of a substance are influenced by its particle size [106]. Cell absorb substances with sizes ranging between 1 and 10 μm [108,109]; thus, nanoparticles owing to their size, easily enter the tissues and are absorbed by cells, ensuring effective drug action at the targeted location with reduced or insignificant side effects [110]. Therapeutically, nanoparticles are used to improve the dissolution, drug targeting, absorption, and breakdown of the enclosed compound and controlled release of the drugs [111].

Zhou et al., in 2017 developed nanoparticles with a dual drug delivery approach for synergistic cancer treatment by encapsulating ascorbyl palmitate (AP) and paclitaxel (PTX) and evaluating their anticancer potential against the B16F10 cell line. Dynamic light scattering and transmission electronic microscopy (TEM) verified the globular profile of the nanoparticles, which had an average diameter of 223 nm and identical zeta potential. In vitro study revealed that AP/PTX-solid lipid nanoparticles (AP/PTX-SLNs) containing AP and PTX in a ratio of 2:1 exhibited an ideal anticancer synergistic effect with improved cellular permeability and uptake of AP and PTX. In comparison, in vivo study revealed that, in B16F10-tumour-bearing mice, AP/PTX-SLNs effectively suppressed tumour growth in the lungs and eliminated the cancer cells more effectively than either of the sole drug-filled SLNs through reduction of the Bcl-2/Bax fraction. In addition, throughout the study with AP/PTX-SLNs, no evident side effects were observed [112].

In 2014, Guney et al., entrapped ascorbic acid (AA) in solid lipid nanoparticles (SLNs) using a hot homogenization technique for effective AA delivery to cancer cells and evaluated their anticancer potential against the H-Ras 5RP7 cell line. Nano Zetasizer ZS and HPLC characterized spherical shapes of AA-SLNs with particle diameters ranging between 50–250 nm and having identical zeta potential. It was found that AA-SLNs released their content more steadily compared to free AA, releasing up to 70% during 96 h as compared to approximately 90% of free AA released in 60 min. At 25 µM/mL concentrations, AA-SLNs exerted maximum cytotoxic effects with 41% cell viability towards H-Ras 5RP7 cells by accumulating up to 68.5% of active caspase-3 content in cells investigated with AA-SLNs as compared to 40.5% active caspase-3 by AA after 72 h. In addition, AA-SLNs exerted no significant cytotoxicity towards control line NIH/3T3 cells [113]. 

Sawant et al., in 2010 prepared liposomal nanoparticles of palmitoyl ascorbate (PA) and evaluated their in vitro and in vivo anticancer potential against murine mammary carcinoma 4T1 cells. Dynamic light scattering (DLS) revealed the 146.6 ± 29.0 nm particle size of the PA liposomes. In vitro study indicated that the generation of superoxide enhanced the anticancer potential of PA liposomes. Additionally, the in vivo study on 4T1 tumour-bearing mice showed that PA liposomes at 20 mg/kg doses suppressed the tumour growth remarkably compared to paclitaxel liposomes [114].

Martins et al., in 2010 prepared ascorbic acid capped nanoparticles of poly-D,L-(lactide-co-glycolide) containing violacein and also evaluated their antitumor activity against leukaemia HL-60 cells. Photon correlation spectroscopy determined the spherical shape of the nanoparticles with diameters ranging from 300 to 550 nm and a solid identical negative Zeta potential. In the violacein release kinetics assay, at the initial 3 hours of analysis, an early burst of nanoparticles was observed, releasing 40–60% of drugs tailed by a steady release of up to 80% after 72 h of analysis. The results of MTT assays showed that the AA-violacein nanoparticles exerted better cytotoxicity against HL-60 cells with an IC_50_ value of 0.2 μM, which was 2.5 times more active than free violacein (IC_50_ = 0.5 μM) [115].

Frungillo et al., in 2009 prepared nanoparticles loaded with trans-dehydrocrotonin (DHC) and L-ascorbic acid 6-stearate (AAS) and evaluated their in vitro antitumoral activity against HL60 cells. Photon correlation spectroscopy determined that their dimensions ranged between 110 to 140 nm with identical negative zeta potential. Kinetics assay of in vitro release revealed that free DHC solubilized after only 6 h of analysis. In comparison, the nanoparticles loaded with AAS and DHC (NP-AAS-DHC) showed a steady release up to 72 h. The cytotoxic effects of NP-AAS-DHC on HL-60 cells were assessed by MTT and Trypan blue exclusion assay, revealing an IC_50_ of 140 and 90 μM, respectively [116].

## 7. Patents as an Anticancer Agent 

In the last few decades, several fundamental discoveries have been transformed into intellectual property with exceptionally realistic opportunities for future utilization. Consequently, new and innovative products and technologies related to the anticancer potential of ascorbic acid and its derivatives were patented. Following are some examples of such patents (Table 1):

## 8. Challenges and Current Status

To utilize ascorbic acid, stability is a major challenge as it is simply degraded on exposure to high pH, aqueous mediums, oxygen, and metal ions. To improve its stability, some modifications have been made to ascorbic acid molecules to generate stable ascorbic acid derivatives such as L-ascorbyl-6-palmitate, L-ascorbic acid 6-phosphate, and L-ascorbic acid 2-glucosidase. Despite being more stable than the parent compound, they do not have direct antioxidant activity and require in vivo enzymatic conversion to L-ascorbic acid. When used topically, these derivatives also have lower permeability through the skin compared to their parent compound, ascorbic acid [111]. 

At the recommended dose, ascorbic acid is safe in healthy individuals; the recommended daily dietary dose of vitamin C is approximately 100 mg. However, in critically ill patients, vitamin C dosing is still a matter of concern [122].

There have been documented cases that daily intake greater than 2 g/day can induce oxalate crystal nephropathy. In some cases, it was also observed that intake as low as 480 mg/day orally or a single dose of 45 mg intravenously can induce oxalate crystal nephropathy [123]. In individuals with a history of kidney stones, amounts greater than 1 g/day significantly raise the risk of kidney stone recurrence [124]. During the metabolic process, ascorbic acid breaks down in tissues to dehydroascorbic acid, then to diketogulonic acid, and is ultimately catabolized to oxalate, which is excreted in urine [125]. The supersaturated oxalate in the tubules favourably deposits as crystals, developing into stones that in turn damage the tubular epithelium [122,123]. Ascorbic acid-induced oxalate nephropathy can lead to severe consequences, including chronic renal disease necessitating long-term dialysis or transplantation, and even death [123]. The duration and dose of ingested ascorbic acid in patients with underlying disease influences the development of acute kidney injury.

Globally, the ascorbic acid market was valued at USD $1273.05 million in 2020, and in the period from 2020 to 2027, it is expected to expand with a compound annual growth rate of 4.6% [126]. As per the report of 2016, the pharmaceutical industry accounted for about 30% of total demand for ascorbic acid [127]. 

## 9. Future Prospects and Conclusions

Cancer is the second leading cause of death worldwide, and numerous approaches as well as materials are being explored to address this deadly disease. In this context, ascorbic acid has recently emerged as a prospective candidate. The potential functions of ascorbic acid covered above are associated with sufficient contact of ascorbate with tumour cells in the tumour environment through effective distribution [59]. Thus, a high dose of ascorbic acid is important for exerting the anticancer effect. However, it should not be viewed as a universal model for cancer treatment [66]. Understanding the pharmacokinetics of ascorbate distribution into the tumour cells is vital for the planning of clinical trials and support for novel ascorbate treatments [59,66], as oral and intravenous administration result in different plasma pharmacokinetics [76]. A number of case studies and clinical trials on cancer patients have described that ascorbic acid at high doses, alone, or in combination with chemotherapies improves the quality of life with less toxicity [128,129]. Vitamin C hinders cancer progression by targeting different vulnerable nodes such as HIF, GLUT1 [46,59,66], and TET [130,131]. For a long period, ascorbic acid and its derivatives have been controversially cited as potential anticancer agents. Wang et al., observed that in a phase 1 clinical study of patients with gastric and metastatic colorectal cancer, a high dose of ascorbic acid with other anticancer agents demonstrated potentially improved efficacy with suitably reduced side effects, enhancing the patient’s quality of life [132]. Lv et al., in their retrospective study on post-surgical hepatocellular carcinoma patients, observed that administration of intravenous ascorbic acid prolonged disease-free survival [133]. Zhao et al., in a clinical study of elderly patients with acute myeloid leukaemia, witnessed that ascorbic acid with other anticancer agents had a better rate of complete remission (CR) and overall survival without any substantial toxicity as compared to the anticancer agents alone [134]. Macan et al., developed two sequences of 6-(1,2,3-triazolyl)-2,3-dibenzyl-L-ascorbic acid analogues, which displayed strong cytostatic activity against breast adenocarcinoma cell lines without any substantial toxicity towards normal fibroblasts [90]. Similarly, Harej et al., developed new 4-substituted 1,2,3-triazole L-ascorbic acid analogues that exhibited the highest antiproliferative activity towards breast adenocarcinoma cells with greater cytotoxicity than one of the reference compounds, carboxyamidotriazole [91].

At present, several preclinical and clinical examinations have pointed to the ability of parenteral ascorbate to act synergistically with chemo or radiotherapy without any interference [135,136,137], in turn enhancing cancer patients’ quality of life by protecting normal tissues from damage caused by chemotherapy [138]. Thus, high-dose intravenous ascorbate and its derivatives signify an inexpensive alternative to anticancer therapy. Moreover, they should be further explored as potential anticancer agents in clinical trials either alone or in combination, given their low toxicity, ready availability, and low cost.

Although the anticancer potential of ascorbic acid was reported a half-century ago, much of its mechanism associated with anticancer activity has remained obscure. However, some findings in recent years have extended our understanding of the biological functions and mechanism of involvement of ascorbate in cancer therapy as an anticancer agent, underlining a range of interesting and promising hypotheses that suggest there is a good basis for using ascorbate in cancer treatment. Ascorbic acid has a critical role in the function of HIFs, TETs, GLUT-1, FIHs, and PHDs, which are fundamentally involved in cancer development and progression. Discovering the pathways regulated by ascorbate will facilitate progress in the development of innovative treatments to sensitize tumours to ascorbic acid use. We have reviewed some of the reported novel ascorbic acid derivatives as anticancer agents, and it was observed that some of the derivatives were comparable to standard drugs used in the experiments with lower toxicity to no toxicity towards normal human cells.

High-dose intravenous ascorbate and its derivatives have the potential to deliver favourable and economical anticancer therapeutic opportunities that should be further explored. Due to their ready availability in nature, low toxicity, and low cost, ascorbic acid and its derivatives could become important therapeutic options in our battle against cancer. However, we will need to pursue conclusive answers on the clinical benefits of ascorbic acid in the treatment of cancer.

## Figures and Tables

**Table 1:** List of some patents related to anticancer activity of ascorbic acid and its derivatives.

S. No	Invention Disclosed	Patent Application Number	References
**1**	A patent was filed to present an invention comprising nucleobase or its derivatives or its analogues and an ascorbic acid molecule or its derivatives or its analogues, attached together with or without oxygen bond in-between to form a single molecule. Once administered, it is expected to exert antiviral and anticancer effect	PCT/IB20 17/057270	[117]
**2**	A patent was filed for the preparation method of novel 3-O-(p-mesylate benzyl)-ascorbic acid as an anticancer agent	CN201710195165A	[118]
**3**	An acylation derivative of L-ascorbic acid, wherein hydroxyl functionality bound to second place carbon of L-ascorbic acid is substituted by a substituent capable of being degraded in vivo and being converted into a hydroxyl group or is unsubstituted, and a hydroxyl group bound to the position 6 of L-ascorbic acid is acylated by an acyl group with a branch	JP2015152439A	[119]
**4**	The patent was filed for a composition of polyphenol and ascorbic acid or its derivatives for aiding in anticancer therapy. Polyphenol with an amount of 50.0–99.9 parts by weight and ascorbic acid or its derivatives in an amount of 0.1–50.0 parts by weight, extraordinarily improved anticancer effects of conventional anticancer agents in contrast to the individual use alone	PCT/KR2004/003478	[120]
**5**	A patent was filed to present the invention of a pharmaceutical preparation comprising L-ascorbic acid derivative with anti-malignant tumour agent, which can be used to treat cancer	EP98106276A	[121]

## Data Availability

The data that support our specific findings in this review are available from the authors upon reasonable request.

## Abbreviations

GLUT	Glucose transporter
HIF	Hypoxia-inducible factor
TET	Ten-eleven translocation
FIH	Factor Inhibiting HIF
PHD	Prolyl hydroxylase domain enzyme
VHL	von Hippel-Lindau
DHA	Dehydroascorbate
NADPH	Nicotinamide adenine dinucleotide phosphate
GSH	Glutathione
GSSG	Glutathione disulphide
NADP	Nicotinamide adenine dinucleotide phosphate
PARP	Poly (ADP- ribose) polymerase
NAD	Nicotinamide adenine dinucleotide
GAPDH	Glyceraldehyde 3-phosphate dehydrogenase
NAM	Nicotinamide
5mC	5-methylcytosine
5hmC	5-hydroxymethylcytosine
5fC	5-formylcytosine
5CaC	5-carboxylcytosine
AML	Acute myeloid leukaemia
CMML	Chronic myelomonocytic leukaemia
Fe^2+^/α-KGDD	Iron/α-ketoglutarate-dependent dioxygenases
ROS	Reactive oxygen species
HNE	4-hydroxynonenal
AscH−	Ascorbate
Asc⋅−	Ascorbate free radical
H_2_O_2_	Hydrogen peroxide
HO⋅	Hydroxyl peroxide radical
MTX	Mitoxantrone
PA	Palmitoyl ascorbate
DOX	Doxorubicin
DC	Docetaxel
EPI	Epirubicin
PTX	Paclitaxel
TEM	Transmission electronic microscopy
SLN	Solid lipid nanoparticle
DLS	Dynamic light scattering
DHC	trans-dehydrocrotonin
AAS	L-ascorbic acid 6-stearate
5-FU	5-fluorouracil
